# Burnout and quality of life among nurses working in selected mental health institutions in South West Nigeria

**DOI:** 10.4314/ahs.v21i3.54

**Published:** 2021-09

**Authors:** Morufat A Alabi, Adeyinka G Ishola, Adenike C Onibokun, Victor O Lasebikan

**Affiliations:** 1 Department of Nursing, College Of medicine, University of Ibadan. Ibadan, Nigeria; 2 Department of Psychiatry, College Of medicine, University of Ibadan. Ibadan, Nigeria

**Keywords:** Nurses, burnout, quality of life, workplace, organizational factors, more-urban

## Abstract

**Background:**

Burnout remains a huge public health problem among nurses.

**Methods:**

A cross-sectional descriptive study assessed 259 nurses from two Neuropsychiatric hospitals in Nigeria. Data was collected using a sociodemographic/ job related questionnaire, the Maslach Burnout Inventory (MBI), and the Short-Form health survey (SF-12). The associations between sociodemographic characteristic and burnout was anaysed using Chi square test, between burnout and quality of life using Spearman correlation statistics. Predictors of burnout were determined using binary regression analysis

**Results:**

Prevalence of emotional exhaustion (EE) was 44.4%, depersonalization (DEP) 31.7% and reduced personal accomplishment was 98.8%. Predictors of EE were: poor funding from management, OR = 0.38 (95% CI 0.15–0.95) and role conflict, OR = 2.44 (95% CI 1.03–5.78), while the predictors of DEP, were age group, 31–40 years, OR = 0.37 (95% CI 0.18–0.77), male gender, OR = 2.55 (95% CI 1.40–4.65), role conflict, OR = 6.53 (95% CI 0.88–7.81) and working at more urban city, OR = 3.07 (95% CI 1.54–6.16). The mean total Quality of life (QOL) scores were significantly higher among respondents who had no EE and DEP p < 0.001.

**Conclusion:**

Burnout is high among mental health nurses and is associated with poor quality of life.

## Introduction

The mental health of nurses is important because of the high level of stress experienced by nurses during their clinical, research, and or administrative work leading to the syndrome of burnout. 1, 2 For example, recently, nurses have had to engage in tedious patients care, yet they have to navigate a rapidly expanding nursing knowledge base, attend to more onerous maintenance of certification requirements and face an unprecedented level of scrutiny to ensure quality nursing care for their patients.^1^

Burnout syndrome is a sustained response to chronic work stress with three dimensions: emotional exhaustion, depersonalization and lack of personal accomplishment.^2^

While the theoretical concept of burnout is grounded in many theories, specifically, an important framework of nurse burnout is the Conservation of Resources Theory (RCT), which focuses on four resources, objects, conditions, personal characteristics and energy.^3^ According to the theory, burnout occurs in nurses as a result of perceived or actual loss of these four resources. Furthermore, nurse burnout could affect work performance, leading to lower alertness and overall quality of care.^4^

A 2001 study reported that 43% of nurses in United States hospitals had emotional exhaustion, while 37% providing direct patient care in nursing homes and 33% of hospital nurses had burnout.^5^ A study of Aiken and co-investigators involving over 43,000 nurses practicing in more than 700 hospitals in five European and North American countries, indicate that the design of nursing work, widespread workforce management problems and inadequate nurse workforce are the most important determinants of burnout among nurses.^6^ A similar observation was noted in a cross countries comparison involving six countries, viz: U.S.A., Canada, U.K., Germany, New Zealand, and Japan, where higher levels of burnout were associated with lower ratings of the quality of care independent of nurses' ratings of practice environments.^7^

In Africa, a cross-sectional survey of 309 female nurses in private and public hospitals in Kenya, Tanzania and Uganda indicated that about a third of the sample had burnout.^8^ In a systematic review of 12 studies from seven African countries, using the Maslach Burnout Inventory, the prevalence of emotional exhaustion was 66%, depersonalization was 37%, and low personal achievement was 49%.^9^

Several studies have been carried out on burnout among nurses in Nigeria. For example, in a study carried out in a General Hospital in Oyo state, 39.1% of the respondents had burnout in the area of emotional exhaustion, 29.2% in the area of depersonalization and 40.0% in the area of reduced personal accomplishment.^10^ In another study, at the University of Nigeria Teaching Hospital, Enugu State, the prevalence of emotional exhaustion was 42.9%, depersonalization 47.6%, and reduced personal accomplishment was 53.8%.^2^

Several factors have been reported to be associated with burnout among nurses, and these include age of the nurse, gender, years of service, designation of the nurse and inadequate personnel.^11^ Others are difficult or demanding patients, inadequate clinical supervision, nurse/nurse conflict, shift duty,^12^ lack of support from the management,^2^ excess workload, unevaluated work, underpayment, leadership style, conflicts with physicians,^13^ lack of social support, presence of stressors related to private life and job insecurity.^10^

The issue of burnout in Nigeria is worrisome, given the serious shortage of nurses in the country. Nigeria has 1.2 nurses per 1000 population. Compared to other Africa countries, it is only Ethiopia that reported a higher nurses shortage of 0.7 per 1000 population compared to Nigeria.^14^ Specifically in the year 2012, it was reported that in Nigeria, there were 136,000 registered nurses, equating 1 per 1176 population,^15^ a situation worse for mental health nurses, given that Nigeria has 0.004 mental health nurses per 1,000 population.^16^ This is inadequate compared with the WHO recommendation of 2.3 health workers per 1000 population and could be a strong pointer to the development of burnout among mental health nurses.^15^

Among mental health nurses, the lack of insight and failure to adhere to nursing instructions makes nurses impinge on the autonomy of their patients, creating substantial ethical dilemmas for the nurses that may further influence the intensity of burnout.^10^ To corroborate this, Prospers noted the difficulties encountered by mental health nurses in tacking complex situations such as verbal and physical aggression, agitation, suicidal behaviour, violence, absconding and voluntary discharge.^17^

Burnout among nurses is a predictor of diagnosable mental disorders such as depression and anxiety disorder,^18^ suicide,^19^ as well as physical health problems such as diabetes,^20^ and cardiovascular diseases.^20^ Burnout among nurses is a predictor of low retention of nurses in the nursing industry,^21^poor quality of life.^22^ Burnout in nurses also affects the quality of nursing care provided, patients recovery rate, high rates of absenteeism and sick leave.^23^

Quality of life (QOL) is defined as health as perceived by the individual.^24^ According to the World Health Organization, QOL has physical, psychological, social and environmental components,^24^ which may be influenced by burnout.^25^ Regarding nurses, it has been shown that QOL is influenced by work and the work environment, of which relationships with supervisors, peers and colleagues are important correlates, which are factors that also lead to burnout.^25^ Several studies have demonstrated the effect of work and work environment on QOL of nurses,^25^–^27^ and have also shown that there seems to be a relation between mental distress and QOL. 28 In Nigeria, several studies have been conducted on burnout among nurses,^2^, ^10^ but there is hardly any published data on burnout and QOL among mental health nurses. Furthermore, how burnout affects the quality of life of nurses is still not clear, despite research findings indicating that quality of life is closely related to professional life and should be assessed in occupational health studies.^29^ Thus, we assessed the prevalence of burnout among mental health nurses in selected psychiatric hospitals in Nigeria as well as their associated factors. We also assessed the relationship between burnout and quality of life (QOL).

## Methods

### Study design/Setting

This was a descriptive cross-sectional study where the sample was drawn from the two Federal Neuropsychiatric Hospitals in South West of Nigeria. They are the Federal Neuropsychiatric Hospital, Aro, Abeokuta (FNPHA), a less urban city, and the Federal Neuropsychiatric Hospital, Yaba (FNPHY), Lagos, a more urban city.

The FNPHA is a 546-bed hospital, established in 1954 and provides inpatient, outpatient, and 24-hour emergency services to mentally ill patients and patients with neuropsychiatric conditions. The hospital currently has 348 nurses. On the other hand, the FNPHY is a 530-bed hospital which was established in 1907. It provides inpatient, outpatient, and 24-hour emergency services to individual living with mental illness and individuals with neuropsychiatric conditions. The hospital has a total of 340 nurses.

### Eligibility

To be eligible for the study, participants were expected to have been working in the hospital for at least 6 months to ensure that they were familiar with the dynamics of day to day activities of the ward.

### Exclusion Criteria

Excluded were nurses who had any pre-existing mental health problem and or chronic general medical conditions.

### Sample Size Estimation

The minimum sample size (n) was obtained using the sample size estimation for a descriptive cross-sectional study.30


$$\text{n}=\frac{\text{z2p (1-p)}}{\text{d}2}$$

where z = standard score corresponding to 95% confidence level (1.96), p = probability that a nurse will have burnout (0.29)10, d = precision expected at 95% CI (0.05).

Thus, $\begin{array}{c} \text{(n) = 1.962}\times \text{(0.29) (1-0.29) = 322}\\ \text{0.052} \end{array}$

Since study population is less than 10,000, finite population correction was done using the formula below:30

$$\text{nF=}\frac{\text{n}}{\text{1+n/N}}$$

where

nF = Desired sample size when the population < 10,000, n = minimum sample size obtained by calculation (322)


$$\text{nF=}\frac{\text{322}}{\text{1 + 322/688}}=214$$

An additional 20% of the desired sample size was added in order to increase precision and also to compensate for those who may not respond.

Thus, the final sample size $\text{nFF=}\frac{\text{nF}}{\text{1 + (2/10)}}=270$

Since the number of nurses working in the two hospitals differs, the participants were recruited in each of the two hospitals by proportional sampling method, 136 from FNPHA and 134 from FNPHY.

### Sampling Technique

The list of all the nurses in each of the two study sites was obtained from the administrative department of the respective hospitals and all the nurses were listed by their wards. The participants enrolled in each department was determined by proportional sampling method. The first participant was randomly selected and subsequent ones systematically selected until all study participants in each of the hospitals were selected.

### Instrument for Data Collection

Three instruments were designed to achieve the objectives of this study, which are:

#### 1. Socio-demographic/ Job related questionnaire

This yielded information on items such as age, sex, marital status, religion, qualification, number of years spent on the job, shifts being run, management support, role conflict and organizational factors/expectation which is defined as increase in renumeration, increase in funding and employment of more Nurse. This questionnaire was adopted from a previous study among nurses in Nigeria.^10^

#### 2. Maslach Burnout Inventory (MBI)

The MBI is a 22-item self-report inventory, designed to measure the characteristics of burnout with 0- Never 1- A few times a year, 2- Many times a year, 3-A few times every month, 4- Many times every month, 5- A few times every week, 6-Everyday. The prevalence of burnout is determined in three areas, emotional exhaustion, depersonalization and personal accomplishment.^31^

**Scoring:** A score of ≥ 27 in items 1 to 9 of the Maslach Burnout Inventory, signifies a high burnout in the area of emotional exhaustion. A score of ≥ 13 in items 10 to 14, signifies a high burnout in the area of depersonalization, while a score of ≤ 33 in items 15–22 signifies a high burnout in the area of personal accomplishment. This is the cutoff point obtained during the validation process in Nigeria.^32^

#### 3. The 12-Item Short Form health survey (SF-12)

The SF-12 is a Health-Related Quality of Life instruments. 33 The SF-12 is a multi-item scale that assesses eight health concepts: 1) physical functioning (addressed by question 3) 2) Role physical (addressed by question 4) 3) Bodily pains (addressed by question 1, 7, 8) 4) General health (addressed by question 1, 11) 5) Vitality (addressed by question 9) 6) Social functioning (addressed by question 6, 10) 7) Role emotional (addressed by question 5) and 8) Mental health (addressed by question 9). Physical functioning, role physical, bodily pains, general health comprises the physical health component (PHC) of SF-12 while vitality, social functioning, role emotional and mental health constitute the mental health component (MHC) of SF-12

**Scoring:** A total mean score is generated for all the items of the SF-12, the higher the mean score, the higher the quality of life of the person assessed.

### Validity of the Instruments for Data Collection

The MBI has cross-cultural reliability and validity^31^ and has been previously used and validated in Nigeria.^32^ In Nigeria, a study aimed at establishing the psychometric properties of the MBI, using a sample of health care workers obtained reliability coefficients as follows: Cronbach's alpha of 0.86 and split-alpha of 0.57. Its validity was also established by its positive correlation with the General Health.^32^ The modified version of the MBI was found to have the same factor structure with the original version.^32^ The SF-12 is the abridged version of SF-36 which has been validated in Nigeria, where the concurrent validity scores for scales and domains ranges between 0.749 and 0.902 with the highest and lowest scores in the General Health (0.902) and Bodily Pain (0.749) scale,^34^ The SF-12 has been previously used in Nigeria,^35^ and has been found to have factorial invariance with the original version.^36^

### Pre-test

A pre-test was carried out among a sample of nurses at the University College Hospital, Ibadan. During this period, all instruments of data collection were pre-tested among 10% of the proposed study population to determine the feasibility, understandability of the research instruments, ability to follow-up the entire research protocol as well as the administration time of all the instruments of data collection.

### Ethical consideration

Ethical approval was obtained by the researcher from FNPHA (PR013/18) and FNPHY (FNPHY/ERC/18/226) and also the joint UI/UCH ethical review committee in line with the Helsinki declaration to ascertain that the methodology does not contravene laid down principles for researches involving human beings and informed consent gained from the participants. Written permission was sought from the authors that validated the MBI before the commencement of the study;^32^ however, permission was not sought for SF-12 which is a generic instrument.

Informed consent: Written informed consent was obtained from the participants and each study participant was given detailed information on the research to be carried out.

### Confidentiality of data

All information given towards the completion of this research work was not disclosed to any unauthorized person. The participants were assured that their identity will be kept in confidence by the investigator. The soft copy of the collected data was pass worded to ensure that unauthorized individuals does not have access to it. The hard copy of the data and other files were kept locked in a safe.

### Method of Data Analysis

For univariate analysis, the prevalence of burnout was illustrated as descriptive statistic. The association between sociodemographic data and burnout was determined using Pearson Chi Square statistic, while the association between burnout and mean QOL scores was determined using the independent t test. The correlation between the dimensions of the MBI and the domains of SF-12 was determined using Spearman correlation statistic, while the predictors of burnout were determined using binary logistic regression. All analyses were set at p < 0.05, 95% CI and done using Statistical Package for Social Sciences version 22.

## Results

**Figure 1:**
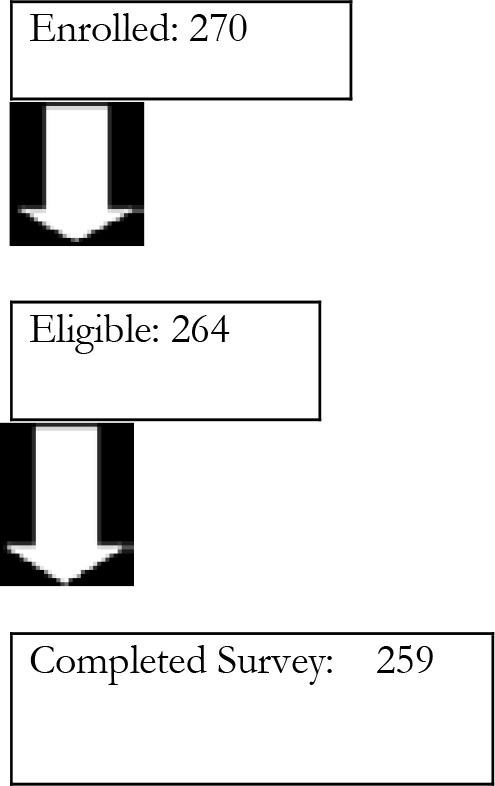
Sample Selection Flow Chart

In this study, 270 participants were enrolled; however, 264 met eligibility criteria, and 259 completed the survey (Figure 1). The mean age of all respondents was 37.97 ± 8.41 years.

### Socio-demographic Characteristics and Burnout

The highest proportion of the respondents (71.4%) was between 31–40 years of age, a higher proportion (69.1%) were female, (73.4%) were currently married, (82.6%) were Christians, while (53.3%) were diploma nurses. The mean age of all respondents was 37.97 ± 8.41 years.

Some, (64.9%) had spent more than 5years on the job, and (25.9%) of the respondent were principal Nursing Officer by rank while (72.2%) of the respondents were currently running shift; and (35.5 %) of the respondent run shift for 15–21 days.

A quarter of the respondents expected the management of the hospital to provide equipment to work with, and half (49%) of the respondent reported that the job placed demands on their private life.

Of the 259 respondents, 115 (44.4%) reported high burnout (EE) and others as shown in Table 1.

**Table 1:** Socio-demographic Characteristics and Burnout

Variables	Emotional Exhaustion				Depersonalization				Personal Accomplishment			
	High (115)	Low (144)	X^2^	P	High (82)	Low (177)	X^2^	p	High (256)	Low (3)	X^2^	p
**Age group**	n (%)	n (%)			n (%)	n (%)			n (%)	n (%)		
21–30years	23 (41.1)	33 (58.9)	0.4	0.8	23 (41.1)	33 (58.9)	6.9	0.03	56 (100)	-	2.0	0.4
31–40 years	54 (44.3)	68 (55.7)			29 (23.8)	93 (76.20			121 (99.2)	1 (0.8)		
>40 years	38 (46.9)	43 (53.1)			30 (37.0)	51 (63.0)			79 (97.5)	2 (2.5)		
**Gender**												
Male	49 (61.3)	31 (38.8)	13.3	< 0.001	37 (46.2)	43 (53.8)	11.4	0.001	78 (97.5)	2 (2.5)	1.82	0.1
Female	66 (36.9)	113 (63.1)			45 (25.1)	134 (74.9)			178 (98.8)	1 (0.6)		
**Marital status**												
Unmarried	32 (46.4)	37 (53.6)	0.2	0.7	28 (40.6)	41 (59.4)	3.5	0.06	69 (100)	-	1.1	0.2
Married	83 (43.7)	107 (56.3)			54 (28.4)	136 (71.6)			187 (98.4)	3 (1.6)		
**Religion**												
Christian	95 (44.4)	119 (55.6)	0.03	0.9	66 (30.8)	148 (69.2)	1.6	0.4	211 (98.6)	3 (1.4)	0.63	0.7
Islam	19 (44.2)	24 (55.8)			16 (37.2)	27 (62.8)			43 (100)	-		
Others	1 (50)	1 (50.0)			-	2 (100)			2 (100)	-		
**Qualifications**												
Diploma	67 (48.6)	71 (51.4)	2.1	0.1	45 (32.5)	93 (67.4)	0.1	0.7	138 (100)	-	3.5	0.06
Degree + diploma	48 (39.7)	73 (60.3)			37 (30.6)	84 (69.4)			118 (97.5)	3 (2.5)		
**Rank**												
Junior Ranks	42 (53.2)	37 (46.8)	3.5	0.06	29 (36.7)	50 (63.3)	1.3	0.2	79 (100)	-	1.3	0.2
Intermediate	73 (40.6)	107 (59.4)			53 (29.4)	127 (70.6)			177 (98.3)	3 (1.7)		
Senior												
**Years in Job**												
< 4 years	34 (37.4)	57 (62.6)	2.8	0.2	39 (42.9)	52 (57.1)	8.1	0.004	91 (100)	0 (0.0)	1.64	0.2
≥ 4 years	81 (48.2)	87 (51.8)			43 (25.6)	125 (74.4)			165 (98.2)	3 (1.8)		
**Run shifts**												
Yes	94 (50.3)	93 (49.7)	9.4	0.002	59 (31.6)	128 (68.4)	0.004	0.9	186 (99.5)	1(0.5)	2.28	0.1
No	21 (29.2)	51 (70.8)			23 (31.9)	49 (88.1)			70 (97.2)	2 (2.8)		
**Organizational Expectation**												
Increase remuneration	36 (65.5)	19 (34.5)	13.3	0.01^BS^	18 (32.7)	37 (67.3)	1.13	0.89	54 (98.2)	1 (1.8)	1.5	0.8
Increase funding	23 (71.9)	9 (28.1)			12 (37.5)	20 (62.5)			32 (100.0)	0 (0.0)		
Funding/remuneration	21 (38.2)	34 (61.8)			18 (32.7)	37 (67.3)			54 (98.2)	1 (1.8)		
Employ more nurses	31 (59.6)	21 (40.4)			14 (26.9)	38 (73.1)			52 (100.0)	0 (0.0)		
No Expectation	33 (50.8)	32 (49.2)			20 (30.8)	45 (69.2)			64 (98.5)	1 (1.5)		
**Private life**												
Not at all	14 (31.1)	31 (68.9)	20.7	<0.001	14 (31.1)	31 (68.9)	7.1	0.07	45 (100)	-	2.72	0.4
Mildly	19 (27.5)	50 (72.5)			14 (20.3)	55 (79.7)			67 (97.1)	2 (2.9)		
Moderately	70 (55.1)	57 (44.9)			49 (38.6)	78 (61.4)			126 (99.2)	1 (0.8)		
Severely	12 (66.7)	6 (33.3)			5 (27.8)	13 (72.2)			18 (100)	-		
**Role conflict on duty**												
Nurse/nurse	18 (46.2)	21 (53.8)	7.9	0.04	20 (51.3)	19 (48.7)	15.5	0.001	39 (100)	-	2.33	0.5
Nurse/doctor	41 (38.0)	67 (62.0)			38 (35.2)	70 (64.8)			106 (98.1)	2 (1.9)		
Nurse/Any other person	41 (57.7)	30 (42.3)			19 (26.8)	52 (73.2)			71 (100)	0 (0.0)		
None	15 (36.6)	26 (63.4)			5 (12.2)	36 (87.8)			40 (97.6)	1 (2.4)		
**Hospital**												
Aro	64 (52.5)	58 (47.5)	6.1	0.01	23 (18.9)	99 (81.1)	17.5	<0.001	122 (100)	-	2.70	0.1
Yaba	51 (37.2)	86 (62.8)			59 (43.1)	78 (56.9)			134 (97.8)	3 (1.2)		

EE was more prevalent in men X2 = 13.3, p < 0.001, among nurses who were running shift X2 = 9.4, p < 0.001, and among nurses working at FNPHA X2 = 6.06, p = 0.01. EE was also more prevalent among nurses who had conflict with other, X2 = 8.0, p = 0.04 (Table 1). Post-hoc pairwise comparison indicates that this significant difference was due to a higher proportion of nurses having nurse/doctor conflict than having conflict with nobody X2 = 5.9, p = 0.01.

Prevalence of DEP was also highest among nurses, 21–30 years compared with older nurses X2 = 6.9, p = 0.03 (Table 1). Post-hoc pairwise comparisons indicate that this was due to a higher prevalence of DEP among nurses 21–30 years of age compared with those between 31 and 40 years (Not Shown in Table 1). DEP was also more prevalent among men X2 = 11.4, p = 0.001 There was also a significant difference in the proportion of nurses with role conflict who had DEP X2 = 15.1, p = 0.001 (Table 1). Post-hoc pairwise comparison indicates that this difference was due to a higher proportion of respondents with nurse/nurse conflict than nurse/any other person conflict X2 = 5.5, p = 0.02 and a higher proportion of respondents with nurse/nurse conflict than having no conflict with anyone X2 = 12.5, p = 0.0004 (Not Shown in any Table). DEP was also more prevalent among nurses at FNPHY compared with FNPHA X2 = 17.5, p < 0.001 (Table 1). There were no sociodemographic correlates of RPA (Table 1).

### Association between Burnout and Quality of Life

In the physical health component of QOL, the mean QOL scores were significantly higher among respondents who had no emotional exhaustion t = -11/5, p < 0.001, no depersonalization t = - 17.0, p < 0.001 but not among those with reduced personal accomplishment t = - 1.6, p = 0.1 (Table 2). There was a negative correlation between MHC and EE p < 0.001, between MHC and Dep p < 0.001, but not between MHC and RPA, but not between MHC and RPA. There was also a negative correlation between PHC and EE p < 0.001, between MHC and Dep p < 0.001, but not between PHC and RPA. Furthermore, there was negative correlation between MHC and EE p < 0.001, between MHC and Dep p < 0.001, but not between MHC and RPA (Table 3).

**Table 2:** Association between Burnout and Quality of Life

	High Burnout		**Physical** **Health**	Low Burnout				
	n	Mean	SD	**n**	Mean	SD	t	p
Emotional Exhaustion	115	14.88	3.43	144	20.17	3.88	-11.5	< 0.001
Depersonalization	82	12.99	2.72	177	20.06	3.27	-17.0	< 0.001
Personal Accomplishment	256	17.78	4.48	3	21.33	7.77	-1.6	0.1
	High Burnout		**Mental** **Health**	Low Burnout				
	n	Mean	SD	n	Mean	SD	t	p
Emotional Exhaustion	115	18.94	3.71	144	20.63	4.21	-3.3	0.001
Depersonalization	82	17.54	3.60	177	20.97	3.83	-6.8	< 0.001
Personal Accomplishment	256	19.87	4.08	3	21.00	4.36	-0.4	0.5
	High Burnout		**Total** **QOL**	Low Burnout				
	n	Mean	SD	n	Mean	SD	t	p
Emotional Exhaustion	115	32.65	5.43	144	37.27	6.43	-6.1	< 0.001
Depersonalization	82	29.70	4.78	177	37.78	5.40	-11.6	< 0.001
Personal Accomplishment	256	35.15	6.37	3	41.00	9.85	-1.5	0.1

**Table 3:** Correlation between Burnout Dimensions and Quality of Life

		Emotional Exhaustion	Depersonalization	Personal Accomplishment	PSC	MSC	Total QOL
Emotional Exhaustion	r	1.000	.194**	-.049	.584**	.256**	.382**
	p	.	.002	.437	.000	.000	.000
Depersonalization	r	.194**	1.000	-.081	.728**	.430**	.597**
	p	.002	.	.191	.000	.000	.000
Personal Accomplishment	r	-.049	-.081	1.000	.048	.012	.060
	p	.437	.191	.	.445	.852	.337
PSC	r	-.584**	-.728**	.048	1.000	.536**	.833**
	p	.000	.000	.445	.	.000	.000
MSC	r	-.256**	-.430**	.012	.536**	1.000	.886**
	p	.000	.000	.852	.000	.	.000
Total QOL	r	-.382**	-.597**	.060	.833**	.886**	1.000
	p	.000	.000	.337	.000	.000	.

### Predictors of burnout

**Predictors of EE were:** poor funding/remuneration by the management, OR = 0.38 (0.15–0.95 and role conflict with any other person, OR = 2.44, 95% CI (1.03–5.78), while the predictors of DEP, were age group 31–40 years, OR = 0.37, 95% CI (0.18–0.77), male gender, OR = 2.55 (1.40–4.65), nurse/any other person conflict, OR = 6.53 (0.88–7.81) and working at FNPHY, OR = 3.07 (1.54–6.16).

## Discussion

### Prevalence of burnout

The findings from this study draw attention to burnout among nurses who form the major nucleus of health care delivery in Nigeria. They are an important clinical population to whom mental health services should also be directed. This is supported by a burnout prevalence of 44.4% in the area of emotional exhaustion (EE), 31.7% in the area of depersonalization (DEP) and 98.8% in the area of personal accomplishment (PA). The 44.4% who reported EE in our study is marginally higher than the 43% who reported EE among a sample of nurses working in the United States, while the 31.7% who reported for DEP was lower than the 37% reported for nurses providing direct patient care in nursing homes in the US.5 Also our figure for EE (44.4%) was lower than the pooled prevalence of EE in a systematic review of 12 studies from seven African countries, where the prevalence of EE was 66%. The prevalence of DEP in the current study (31.7%) is also lower than the 37% reported in the systematic review. However, we obtained a prevalence of 98.8% for RPA which is twice the 49% reported in the systematic review.^9^

Nevertheless, the rates obtained in the current study are higher than those reported in a study in south eastern Nigeria.^2^ In that, the prevalence of burnout in the area of emotional exhaustion was 42.9%, and 47.6% in the area of depersonalization. In the area of reduced personal accomplishment, the prevalence was 53.8%. In an earlier study in a general hospital in south western Nigeria, 39.1% of the respondents had burnout in the area of emotional exhaustion, 29.2% in the area of depersonalization and 40.0% in the area of reduced personal accomplishment.^10^ By implication, the prevalence of burnout among nurses in Nigeria seems to be increasing with time. This could be due to several factors such as socioeconomic challenges and a deteriorating health care system in Nigeia. For example, Norlund and colleagues found socioeconomic factors to be a predictor of burnout,^37^ and there are indications that the economy of Nigeria is worse off than in previous decades.^38^ Also, the health profile is worse with Nigeria ranking 187 in overall health attainment out of the 191 member states of the World Health Organization.^39^

### Sociodemographic and job-related iorrelates of burnout

In this study, both univariate and multivariate analyses showed that a high proportion of men reported emotional exhaustion and depersonalization. This is supported by results of a recent meta-analysis in a study of oncology nurses.^40^ However, our results findings contradict findings from previous studies, though in other settings both in Nigeria,^2^, ^10^ and in developed countries,^41^ where burnout was more prevalent in female nurses. Thus, the idea that female nurses have higher stress and be at a greater risk of burnout as a result of conflict between traditional gender roles and professional practice is not supported by our findings. Nonetheless, in support of a previous study in Nigeria, 10 we found that shift duties were associated with emotional exhaustion. Other studies have shown that frequent shift duties are associated with increased night shifts, sleep disturbances and longer work hours even among non-health professionals.^42^ This situation may be worrisome among mental health nurses who have to deal with persons with behavioural problems, including violence.^43^

Also, salient was the association between nurses who had spent long on the job and depersonalization, an observation previously reported in Nigeria.^10^ By implication, the current group of mental nurses had not developed positive adaptation which, in a way, is a measure of resilience. This group of nurses may require follow-up because they are likely be at risk of developing psychopathology.^44^

We also found that organizational factors, such as high level of expectation from nurses regarding funding, were associated with emotional exhaustion, an observation that had been noted by previous studies,^10^,^45^. Indeed, a study in Europe, also found that organizational factors were also associated with depersonalization and reduced personal accomplishment.^45^

In the present study, we found that nurse/other person conflict was a significant source of emotional exhaustion and depersonalization, a finding supported by previous studies.^10^ Indeed, the significance role ambiguity plays in the development of burnout among nurses had been emphasized.^46^ This is an issue in organizations where staff do not receive adequate and clear-cut job descriptions. It is often present among nurses with a heavy workload and family responsibilities leading to work-family role conflict.^47^ In fact, our result indicate that the source of major conflict among our sample was nurse/any other person conflict.

We also found a differential pattern in emotional exhaustion and depersonalization across the two settings, with emotional exhaustion more prevalent at FNPHA and depersonalization at FNPHY, a finding previously reported.^48^ This may suggest differential demographic, work-related and organizational differences between the two hospitals as reported in our previous paper.^48^

### Burnout and quality of life

In terms of burnout and quality of life, our analysis shows that nurses with low burnout in the areas of emotional exhaustion and depersonalization had significantly higher QOL in the physical health domain, the mental health domain and the total QOL. There was also a negative correlation between the two domains of QOL, total QOL versus emotional exhaustion and depersonalization respectively. This is expected given than a high level of burnout predicts physical illnesses and mental illnesses.^49^

Our findings suggest that quality of life and burnout among our sample may be determined by the intrinsic characteristic of the job of mental health nurses. As previously suggested, it is possible that quality of life may be driven by the nature and the organization of their job 29. Based on this reflection, our results are a concern and indicate the need for interventions to help mental health nurses suffering from burnout. There is, therefore, need for preventive measures directed to this group of health workers suffering from burnout and those disengaged.

## Strength and limitation of the study

The main limitation of our study was its cross-sectional design. Future design should include possible intervention program for burnout. The use of SF-12 in assessing the quality of life of our current sample may have limited conclusions about specific aspects related to work. The potential for recall bias in the self-reported measures was also a limitation. Individuals with high negative affectivity may perceive their work context more negatively, which would artificially strengthen the associations between burnout symptoms and work environment.

## Conclusion

Burnout in the area of EE was associated with poor funding from management and with role conflict, while burnout in the area of depersonalization was associated with a younger age group, male gender, role conflict, and working in at more urban areas. Burnout also has a negative relationship with QOL.

Workplace screening and intervention for burnout should be made available for this population of health care workers.

## Figures and Tables

**Table 4:** Predictors of Burnout

Variables (Prediction 67.6%)	Adjusted OR	95% CI	p-value
**Emotional Exhaustion**			
**Gender**			
Male	1.75	0.87–3.51	0.12
Female	1		
**Run shifts**			
Yes	0.65	0.22–1.92	0.43
No	1		
**Role conflict**			
Nurse/nurse	1.44	0.53–3.92	0.48
Nurse/doctor	1.01	0.44–2.33	1.0
Nurse/Any other person	2.44	1.03–5.78	0.04
None	1		
**Organizational Expectation**			
Increase remuneration	0.91	0.34–2.47	0.86
Increase funding	1.27	0.39–4-14	0.68
Funding/remuneration	0.38	0.15–0.95	0.04
Employ more nurses	0.92	0.35–0.45	0.87
No Expectation	1		
**Job demand on your private life**			
Not at all	2.64	0.61–11.33	0.19
Mildly	3.56	0.93–13.68	0.06
Moderately	1.32	0.38–4.53	0.66
Severely	1		
**Hospital**			
Aro	0.67	0.33–1.35	0.26
Yaba	1		
**Depersonalization**			
**Age Group**			
21–30years	0.38	0.14–1.08	0.07
31–40 years	0.37	0.18–0.77	0.008
>40 years	1		
**Gender**			
Male	2.55	1.40–4.65	0.002
Female	1		
**Years spent on job**			
< 4 years	1.85	0.82–4.18	0.14
≥ 4 Years	1		
**Role conflict on duty**			
Nurse/nurse	2.17	0.98–4.82	0.057
Nurse/doctor	2.36	0.97–5.74	0.059
Nurse/Any other person	6.53	1.97–21.64	0.002
None	1		
**Hospital**			
Aro	1		
